# 

**Published:** 1898-03

**Authors:** 


					﻿Southern Medical Record
A Monthly Journal of Medicine and Surgery.
Vol. XXVIII.
ATLANTA, GA., MARCH, 1898.
No. 3'
BERNARD WOLFF, M. D., Editor.
Associate Editors :
A. W. GRIGGS, M. D.
j. McFadden gaston. m. d.
WILLIS F. WESTMOREL IND, M. D
B. M. ZETTLER, Business Manager.
Entered at the Post-office at Atlanta, Ga., as Second-class Matter.
The Eoote & Davies Co., Atlanta, Ga.
				

